# Preliminary Classification of Mineral Springs

**Published:** 1916-12

**Authors:** Felix Von Oefele

**Affiliations:** New York


### Preliminary Classification of Mineral Springs.

Dr. FELIX VON OEFELE, New York.

The comparison basis for a medical water must be the good average water. Average water taken from a river or spring contains a certain percentage of solid matter of exclusively invalid character for mankind.

The American balneology is forced to consider the usual European classifications of mineral springs and must adapt where the European classification does not fit for American conditions. The European balneology had a historical development collecting the balneological experience of many thousand years. Exclusively observations of the small European continent have been collected. England, France, Germany and Austria are well known. Russia contains many valuable mineral springs, for instance, in the Caucasus. They are little known by the civilized world. Italy and Spain are again little known. Some time ago the writer studied the mineral springs of Roumania theoretically, compiled by a pupil of Minovici. They are not included in the area of scientific European balneology. The European arrangement of balneological systematic can not be complete. The conditions of the entire large United States do not fit in this small European systematical arrangement.

Very good drinking water may contain 0.04 to 0.05% of dissolved invalid minerals. Common spring water used for domestic drinking purposes must be below 0.1% of mineralization.

These waters usually contain as their main constituents Calcium, Magnesium with small amount of Alkalies, trace of Iron, Silica, Sulfurtrioxyde, Carbondioxyde and Chlorine. Other constituents may be present only as traces. The relationship of these constituents varies very much in different waters.

Every principal classification of mineral springs must be based on the amount and nature of the dissolved natural salts. This classification must show the relationship of the mineral constituents to the human body and particularly to the human serum. The invalid minerals mentioned above are present in an adequate amount in the human serum. This presence within the human serum renders them invalid. Again on the contrary, the ubiquity of the invalid constituents of natural waters enforced the serum of human and other beings to keep the invalid constituents in the dissolved stock of the body by adaptations.

Waters containing below 0.04% total solids are submineralized waters, 0.04-0.05% are low average mineralized, 0.05-

0.1% are high average mineralized and more than 0.1% total solids are supermineralized waters.

Carbonic acid, Sodium, Calcium, Magnesium, Chlorine, and Sulfuric acid are the common invalid constituents of every mineral water. These or their salts alone determine the character of a supermineralized water, if they amount to more than 65% of the entire total solids and if not a valid constituent of the same chemical family characterizes the water by its percentage present. Where no content exceeds 65%, a mixed name is given and every one of these invalid contents, the amount of which exceeds 25% of total solids takes place in the classification.

Potassium, Lithium, Rubidium, Cassium may be present as valid constituents together with prevalent Sodium. If such a valid constituent reaches a certain individual percentage of Sodium and if Sodium were name forming, this valid constituent would replace Sodium within the denomination. The condition is similar where Fluorine or Bromine or Iodine replaces the denominating Chlorine valid constituents, as far the respective element family contains an invalid member.

Other elements besides the element families of invalid common constituents may give medicinal value to mineral springs. Iron, Arsen, Nitric acid, Silicic acid and Phosphoric acid are examples. These or the above valid constituents of medicinal value may be present in predominant amount particularly in submineralized or average mineralized waters. They may also furnish the medical classification of mineral springs. Springs which are really and absolutely characterized by valid constituents are usually submineralized or at least low mineralized.

Some springs unknown in Europe or United States may not seem to fit into these classes, and may have exceptional representatives in foreign countries, or may be useless for the medical profession. We will therefore not go into theoretical classes without really existing springs, but a reasonable adaptation will not be difficult also for such springs.

### AMERICAN AREAS OF MINERAL SPRINGS.

Mineral springs are known over the entire United States. They have to be arranged by scientific or practical means. This is possible in different ways: 1) in orographical sense by the mountain systems, 2) in geological sense by the stratigraphic condition of the surface, 3) by geological faults and systems of faults in regard to the influence of different sources of magmatic waters, 4) in historical sense by districts of cultural developments where the old Indian districts of culture are still permanent for modern communities of interests, and 5) in political sense according to the arrangement of modern states and counties.

Every one of these five arrangements has some influence upon the systematic arrangement of the following Balneology.

The United States of North America has to be divided into areas and subdivided into districts and fields for balneological research by orographical and geological means. The individual springs have to be located by states, counties and townships.

The coarse divisions are the areas based on the orographical subdivisions of North America. With this end in view then, the principal divisions are: the Atlantic areas, Eastern Mississippi area, Western Mississippi area, decline area of Rocky Mountains, Mountain area and Pacific area.

The decline of the eastern mountain system to the Atlantic Ocean constitutes the Atlantic areas. The Atlantic areas must be subdivided by practical means on account of the concentrated population of the eastern shore into three separate areas: the Middle Atlantic area, the South Atlantic area and the New England area.

The Atlantic areas include with few exceptions the area of the 13 original states of the Declaration of Independence. The eastern declines of the Appalachian Mountains and the Adirondacks were first settled and are still the next largest cities. The mineral springs of these areas were the first to become known and to be visited. They were first developed by adaptations for white settlers. For the last fifty years they have been neglected and have been unable to successfully compete with the European mineral springs.

The Middle Atlantic Area is the middle part of the decline where the ribbons from the oldest to the newest deposits are relatively regularly arranged from the summit of the Appalachian Mountains down to the seashore. The triboundary point of Vermont, Massachusetts and New York is the most northern and Frederiksburg, Virginia is the most southwestern point. It includes the Catskills and the southeast of New York, the states of New Jersey, Delaware, the east of Pennsylvania, the east of Maryland, the east of Virginia and a small northeastern part of North Carolina.

The South Atlantic Area is less regularly arranged and starts from the point where the cretaceous deposits approach the seashore. The arrangement is still in geological ribbons but with a great many of the connecting links missing. It includes a southern triangle of Virginia and the principal parts of North Carolina, South Carolina, Georgia and Florida with the exception of a narrow northwestern ribbon.

The New England Area is arranged in a geological sense very irregularly. It includes Maine, New Hampshire, Massachusetts, Rhode Island, Connecticut, the principal part of Vermont and a very small field of southeastern New York.

The three Atlantic areas yield waters of low mineralization and low temperature.

The Eastern Mississippi area is formed by the decline of the eastern mountains to the central depression of the United States. It includes the northwestern corner of Vermont, the principal part of New York, western Pennsylvania, Kentucky, Tennessee, Alabama, Mississippi and a narrow western ribbon of the states included in the South Atlantic Area. Thermal waters and highly carbonated waters are found on the boundary of the Atlantic areas. The mineral waters of the eastern Mississippi area usually show a higher mineralization. Sodium chlorides are found in few fields. Increased amounts of sulfates are very common constituents of this area. The sulphur culminates in rich intrusions of elementary sulfur on the mouth of the Mississippi.

The Western Mississippi Area is the western plains. The average amount of total solids contained in mineral springs is still lower than in the Eastern Mississippi Area.

The Area of Rocky Mountains decline includes the eastern part of the Rocky Mountains and their eastern decline. The most western three areas of the United States are richer in valuable mineral springs than the eastern areas. But the crowd of visitors of mineral spring resorts comes from the large eastern cities. The western mineral springs are too distant for them for an average common mineral spring cure of four weeks. The high amount of thermal springs must be mentioned especially.

The Western Mississippi Area and the Area of Rocky Mountains decline include the principal parts of the states of Wisconsin, Minnesota, North Dakota, South Dakota, Arkansas, Oklahoma, Louisiana and Texas.

The Mountain Area includes the districts from the Rocky Mountains to the Western Mountains with the principal part of Montana, Wyoming, Idaho, Colorado, Utah, Nevada, New Mexico and Arizona. The waters are commonly rich in alkali salts. Potassium shows commonly a high percentage compared to sodium.

The Pacific Area includes the decline to the Pacific seashore with the principal part of Alaska, Washington, Oregon and California. The mineral springs correspond to springs of active volcanic districts of the old world.

The different properties of the American springs and wells depend upon the local geological and petrographical condition. The mineral springs of the same geological system of a single area belong mostly to one, two or few fields of a uniform classification regarding amount and kind of the dissolved minerals. In some cases the comparison must be extended to similar springs of similar geological or petrographical conditions of other areas and also of other countries. Geological basis and chemical analysis must be considered for the different groups of classification of mineral springs, but also for medical usefulness.

To explain the different kinds of rocks, we will use only the gross divisions of geology i.e. groups and systems. Sections, stages and strata of the systematic geology will be mentioned only very exceptionally. It is a fact that for instance many mineral springs of the upper states of New York are yielded by strata of the same geological age known for Wiesbaden, Ems, Karlsbad and many other famous springs in Germany. Other geological systems are very poor in useful springs in Europe as well as in America.

The crystalline rocks are the oldest geological group without trace of organic residues. Next come the proterozoic, then the palaeozoic group including Ordovician, Devonian, and Silurian systems besides others, then the mesozoic group including triassic and cretaceous systems and then the kaenozoic group including oligokaen and pleistokaen system.

Three isolated parts of present North America rose above water in the beginning of solidification of the earth's surface. There was a large shield of continent in Eastern Canada, southeast of the Adirondack-Appalachian island and a nucleus of Wisconsin island. An emerged peninsular mountain chain was an early palaeozoic product from Wisconsin to Ozark. The system of the Rocky Mountains was the following geological emersion of later mesozoic or early Kaenozoic period. The most recent elevation is the mountain system of the Pacific Coast. Original Mediterranean seas separated the newly emerged mountain systems, but later became solidified plains.

---

**Erysipelas Treated With Diphtheria Antitoxin.** Koller, Correspondenzblatt fur Schweizer Aertzte, July 8, reports his second case successfully treated. 3000 units were injected with relief, subjective and objective in 24 hours but 1000 units more were injected on account of tenderness. The method is ascribed to Pollak, 1914.

---

**Niece Acetone Test.** This is a modification of the familiar Lange test. Ammonium nitrate 30. and sodium nitro-prussid 2. are dissolved in 80 c.c. of water. Mix 1/2-1 c.c. of reagent with 5 c.c. urine. Overlay with 3 c.c. of strong ammonia water. A purple color indicates acetone. Med. Times, Oct.

